# Oncostatin M Mediates STAT3-Dependent Intestinal Epithelial Restitution via Increased Cell Proliferation, Decreased Apoptosis and Upregulation of SERPIN Family Members

**DOI:** 10.1371/journal.pone.0093498

**Published:** 2014-04-07

**Authors:** Florian Beigel, Matthias Friedrich, Corina Probst, Karl Sotlar, Burkhard Göke, Julia Diegelmann, Stephan Brand

**Affiliations:** 1 Department of Medicine II, University-Hospital Munich-Grosshadern, Ludwig-Maximilians-University Munich, Munich, Germany; 2 Institute of Pathology, Ludwig-Maximilians-University Munich, Munich, Germany; 3 Clinic for Preventive Dentistry and Parodontology, Ludwig-Maximilians-University Munich, Munich, Germany; Cincinnati Children's Hospital Medical Center, University of Cincinnati College of Medicine, United States of America

## Abstract

**Objective:**

Oncostatin M (OSM) is produced by activated T cells, monocytes, and dendritic cells and signals through two distinct receptor complexes consisting of gp130 and LIFR (I) or OSMR-β and gp130 (II), respectively. Aim of this study was to analyze the role of OSM in intestinal epithelial cells (IEC) and intestinal inflammation.

**Methods:**

OSM expression and OSM receptor distribution was analyzed by PCR and immunohistochemistry experiments, signal transduction by immunoblotting. Gene expression studies were performed by microarray analysis and RT-PCR. Apoptosis was measured by caspases-3/7 activity. IEC migration and proliferation was studied in wounding and water soluble tetrazolium assays.

**Results:**

The IEC lines Caco-2, DLD-1, SW480, HCT116 and HT-29 express mRNA for the OSM receptor subunits gp130 and OSMR-β, while only HCT116, HT-29 and DLD-1 cells express LIFR mRNA. OSM binding to its receptor complex activates STAT1, STAT3, ERK-1/2, SAPK/JNK-1/2, and Akt. Microarray analysis revealed 79 genes that were significantly up-regulated (adj.-p≤0.05) by OSM in IEC. Most up-regulated genes belong to the functional categories “immunity and defense” (p = 2.1×10^−7^), “apoptosis” (p = 3.7×10^−4^) and “JAK/STAT cascade” (p = 3.4×10^−6^). Members of the SERPIN gene family were among the most strongly up-regulated genes. OSM significantly increased STAT3- and MEK1-dependent IEC cell proliferation (p<0.05) and wound healing (p = 3.9×10^−5^). OSM protein expression was increased in colonic biopsies of patients with active inflammatory bowel disease (IBD).

**Conclusions:**

OSM promotes STAT3-dependent intestinal epithelial cell proliferation and wound healing *in vitro*. Considering the increased OSM expression in colonic biopsy specimens of patients with active IBD, OSM upregulation may modulate a barrier-protective host response in intestinal inflammation. Further *in vivo* studies are warranted to elucidate the exact role of OSM in intestinal inflammation and the potential of OSM as a drug target in IBD.

## Introduction

Oncostatin M (OSM) is a member of the IL-6 cytokine family, which also includes IL-6, IL-11, leukemia inhibitory factor (LIF) and cardiotrophin-1. OSM is mainly produced by activated T cells, monocytes, and dendritic cells, and shows 27% amino acid identity with LIF. The genes for both cytokines lie in close proximity on chromosome 22q12. In a genome-wide association study, a single nucleotide polymorphism (rs2412970) on chromosome 22 within proximity of LIF and OSM has recently been identified as a susceptibility gene for Crohn's disease (CD) [1]. IL-6 is a proinflammatory cytokine up-regulated in chronic inflammatory diseases like inflammatory bowel disease (IBD), and a monoclonal antibody against IL-6 (tocilizumab) is used in patients with rheumatoid arthritis and in clinical studies for the treatment of IBD [2]. IL-6 plays a pivotal role in development, immune regulation, cell survival, growth and homeostasis [3]. gp130 (also called CD130) is the shared receptor subunit for all members of the IL-6 family of cytokines [4]. Two different OSM receptors have been discovered so far: OSM type I, which consists of gp130 and the LIF receptor (LIFR), and OSM type II which consists of gp130 and OSMR-β [5]. Upon ligand binding to these receptor complexes, various signal cascades are activated, including JAK/STAT- and MAP kinase pathways [6], [7]. While gp130 is ubiquitously expressed, the specific OSM receptor subunits OSMR-β and LIFR are confined to certain tissues, including the kidney, pancreas, liver and the lung [6].

OSM plays an important role in hematopoiesis, immunity, bone remodeling, and inflammation [7]. However, the exact role of OSM in inflammation remains to be defined. There are several studies demonstrating both pro- and anti-inflammatory effects of OSM, e.g. inhibiting IL-1β secretion [8] vs. potentiating the effects of IL-1β [9] in synovial fibroblasts. The inflammatory response is mainly mediated via overexpression of acute phase proteins in the liver [10], [11]. Currently, there are only few data regarding regulation of the OSM receptor expression and its detailed signal transduction and specific biological functions in intestinal epithelial cells (IEC) and intestinal inflammation. A previous study showed that adenoviral vector transfer of OSM ameliorated dextrane sodium sulfate (DSS)-induced colitis in mice compared to IL-6, implicating a protective role for OSM in intestinal inflammation [12]. However, when the authors used a different, T_H_2-driven colitis model in mice (oxazolone), viral vector transfer of OSM enhanced the pathology [12]. Therefore, our main study aims were the analysis of OSM-mediated signaling and biological functions in IEC. Our study reveals that OSM may promote intestinal barrier functions via enhanced STAT3-dependent cell proliferation and wound healing as well as upregulation of SERPIN proteins. Increased OSM expression in colonic biopsy specimens of patients with active IBD indicates an important role in human intestinal inflammation. This study contains the first detailed analysis of OSM-induced target genes in IEC and of OSM expression in human IBD.

## Methods

### Ethical Statement

The study was approved by the Ethics committee of the Ludwig-Maximilians-University Munich, Department of Medicine, Munich-Grosshadern (project-nr. 343-09) and adhered to the ethical principles for medical research involving human subjects of the Helsinki Declaration. All patients gave written informed consent prior to colonic biopsy sampling.

### Reagents

Recombinant human OSM, IL-22 and TNF-α were purchased from R&D Systems (Minneapolis, MN, U.S.A.). Antibodies were from BD Transduction Laboratories, Franklin Lakes, NY, U.S.A. (pSTAT1), Upstate Biotechnology, Lake Placid, NY, U.S.A. (pSTAT3), and Santa Cruz Biotechnology, Santa Cruz, CA, U.S.A. (OSM, OSMR-β, LIFR, gp130, STAT1, STAT3, actin, PCNA). Horseradish peroxidase conjugated secondary antibodies to mouse or rabbit IgG and chemiluminescent substrate (SuperSignal West Dura Extended Duration Substrate) were from Pierce (Rockford, IL, U.S.A.). Polyclonal antibodies to phosphorylated janus-kinase-2 (JAK-2, Tyr221), extracellular signal-regulated kinase (ERK)-1/2 (Thr183/Tyr185), phosphorylated stress-activated protein kinase (c-Jun N-terminal kinase) SAPK/JNK-1/2 (Thr183/Tyr185), and phospho-Akt (Ser473) were purchased from Cell Signaling (Beverly, MA, U.S.A.). Anti-ERK-1/2, anti-SAPK/JNK-1/2, and anti-Akt antibodies were also from Cell Signaling (Beverly, MA, U.S.A.). Wortmannin and PD98059 were from Tocris Bioscience (Bristol, UK). Horseradish peroxidase linked anti-rabbit secondary antibody was purchased from Amersham (Arlington Heights, IL, U.S.A.). STAT3- and control siRNA were purchased from Ambion/Life Technologies (Darmstadt, Germany).

### Cell culture

The human colorectal cancer-derived IEC lines SW480, Caco-2, HT-29, HCT116, and DLD-1 were purchased from American Type Culture Collection (Rockville, MD, U.S.A.). Cells were grown in Dulbecco's modified Eagle medium, high glucose (PAA, Pasching, Austria), with 100 IU/mL penicillin, 100 µg/mL streptomycin, and 10% heat-inactivated fetal calf serum (PAA, Pasching, Austria) in a humidified 5% CO_2_ atmosphere at 37°C.

### Reverse transcriptase polymerase chain reaction (RT-PCR), quantitative PCR and microarray analysis

For quantitative PCR, total RNA was isolated using Qiagen RNeasy Kit from Qiagen (Hilden, Germany). RNA concentration and purity was measured on the NanoDrop ND-1000 spectral photometer (Peqlab, Erlangen, Germany) and RNA was reverse transcribed with the Roche Transcriptor First Strand cDNA synthesis Kit (Roche, Mannheim, Germany). Real-time qPCR was performed on a LightCycler480 with SYBR Green PCR Master Mix from Roche. Gene expression was normalized to β-actin in the respective samples. Oligonucleotide primer pairs (Eurofins MWG Operon, Ebersberg, Germany) were designed according to the published sequences avoiding amplification of genomic DNA and are listed in Table 1.

**Table 1:** Primers used for PCR and qPCR.

Gene	Primer combination
gp130 forward	5′-TCAACTTGGAGCCAGATTCC-3′
gp130 reverse	5′-CCCACTTGCTTCTTCACTCC-3′
LIFR forward	5′-ATGGGAAGACATTCCTGTGG-3′
LIFR reverse	5′-CGCAAGACCAGGTGGTAACT-3′
OSMR-beta forward	5′-GGAATGTGCCACACACTTTG-3′
OSMR-beta reverse	5′-ACATTGGTGCCTTCTTCCAC-3′
OSM forward	5′-GCTGCTCAGTCTGGTCCTTG-3′
OSM reverse	5′-CCCTGCAGTGCTCTCTCAGT-3′
SERPINA3 forward	5′-ACACAGGCAATGCCAGCGCA-3′
SERPINA3 reverse	5′-CCTGGCCCCTGTGATCCCTGA-3′
STAT1 forward	5′-TCGGCAGCAGCTTAAAAAGT-3′
STAT1 reverse	5′-CACCACAAACGAGCTCTGAA-3′
STAT2 forward	5′-GCTTCCTCTATCCCCGAATC-3′
STAT2 reverse	5′-TTGCAGTTCATCCACCTGTC-3′
STAT3 forward	5′-AGCTGCACCTGATCACCTTT-3′
STAT3 reverse	5′-AATTGGGGGCTTGGTAAAAA-3′
STAT4 forward	5′-AAGGAACGGCTGTTGCTAAA-3′
STAT4 reverse	5′-CCCCTTTCTGTTGGTCTTGA-3′
STAT5A forward	5′-CAAGGAGAACCTCGTGTTCC-3′
STAT5A reverse	5′-AGTCAAACTTCCAGGCGATG-3′
STAT5B forward	5′-CAACAGGCCCATGACCTACT-3′
STAT5B reverse	5′-TGCTTGATCTGTGGCTTCAC-3′
STAT6 forward	5′-TTGGCTTCATCAGCAAACAG-3′
STAT6 reverse	5′-GGTCCCTTTCCACGGTCA-3′
GAPDH forward	5′-CGGAGTCAACGGATTTGGTCGTAT-3′
GAPDH reverse	5′-AGCCTTCTCCATGGTGGTGAAGAC-3′
β-actin forward	5′-CCTCGCCTTTGCCGATCCGC-3′
β-actin reverse	5′-CCACCATCACGCCCTGGTGC-3′

For microarray experiments, HCT116 cells were starved overnight with medium containing 1% FCS after reaching 70% confluency. On the next day, cells were stimulated in quadruplicates with 100 ng/mL OSM or left unstimulated. RNA was isolated 6 hours after stimulation and RNA concentration and purity was measured. For the analysis of the OSM-induced gene expression, the Agilent Whole Human Genome Oligo Microarray was used in combination with a One-Color based hybridization protocol. Signals were detected with the Agilent DNA Microarray Scanner. Differential gene expression was identified by applying biostatistics using the GeneSpring GX 10 analysis software (Agilent Technologies, Santa Clara, CA, U.S.A.) to normalize and analyze the raw data. OSM-induced gene expression was calculated in comparison to unstimulated cells at the same time point. Welch's approximate t-test (“unpaired unequal variance”, parametric) was applied to the comparison of the different groups. Resulting p-values were corrected for multiple testing using the algorithm of Benjamini et al. [13]. Functional analysis (categories of biological processes, molecular functions and pathway categories) of induced and repressed genes was performed using analysis tools from the Panther homepage [14]. By comparing OSM-regulated gene identification numbers to the distribution of all gene identification numbers represented on the Whole Human Genome Oligo Microarray (Agilent Technologies), it was calculated whether a specific class is over- or underrepresented. P-values of p<10^−5^ (based on binomial test) were considered as a sign of enrichment in the context of a Panther analysis for biological processes, molecular functions and pathway categories. All microarray data presented are MIAME compliant and the raw data have been deposited in a MIAME compliant database in Gene expression omnibus (GEO accession number GSE53295).

### siRNA transfection

HCT116 cells were transfected with siRNA using Lipofectamine 2000 (Ambion/Life Technologies, Darmstadt, Germany) and specific siRNA against STAT3, LIFR and OSMR or an unspecific control siRNA according to the manufacturers' recommendation.

### Signal transduction experiments and immunoblotting

The signal transduction experiments were performed in overnight serum-starved IEC lines as indicated. Cells were stimulated with 100 ng/mL OSM. They were solubilized in lysis buffer containing 1% Nonidet P-40, 20 mM Tris-HCl (pH 7.4), 150 mM NaCl, 2 mM EDTA, 2 mM EGTA, 10 µg/mL aprotinin, 2 mM phenylmethylsulfonyl fluoride, 10 µg/mL leupeptin and phosphatase inhibitors (400 mM sodium orthovanadate and 4 mM NaF) and were passed several times through a 21G needle. After 30 minutes on ice, lysates were centrifuged at 10.000 g for 20 minutes. The protein concentration of each sample was quantified by the Bradford method. Immunoblotting was performed as previously described [15].

### Cell proliferation assays

HCT116 cells were seeded onto 96 well plates and were allowed to attach overnight. Cells were then stimulated with 100 ng/mL OSM, or with cytokine-free medium (negative control) for the indicated time intervals. Cell proliferation was determined by the WST-1 (water soluble tetrazolium) assay (Roche, Mannheim, Germany) and CellTiterGlo assay (Promega, Madison, U.S.A.) according to the manufacturer's instructions. The WST-1 assay quantifies a combination of cellular proliferation, viability, and cytotoxicity; the CellTiterGlo assay is a luminescence cell viability assay which measures ATP of metabolic active cells in a luciferase reaction to determine the amount of viable cells. For each experiment, 16 wells were analyzed.

### Wounding assay

Wounding assays were performed as previously described [16]. Briefly, HCT116 cells were grown in 6 well or 96 well plates to complete confluency. Using a sterile pipet tip, circular wounds were created in each well. Detached cells were removed by washing with PBS, and the cell medium was changed from 10% FCS containing medium to 1% FCS containing medium. The cells were stimulated with OSM (100 ng/mL) or left unstimulated. The next day, the overgrown area was calculated under a confocal laser scanning microscope 510 (Zeiss, Jena, Germany) using AxioVision 4.6 (Zeiss, Jena, Germany). For each group (OSM stimulated and medium stimulated), 16 or more wells were analyzed.

### Apoptosis assays

Similar to cell proliferation experiments, HCT116 cells were seeded onto 96 well plates at a density of 10^5^ cells/well and were allowed to attach overnight. Cells were then stimulated with 10 or 100 ng/mL OSM or with cytokine-free DMEM medium containing 1% FCS (negative control) for 6 hours. Apoptosis was measured using the caspase-3/7 chemoiluminescence assay (Promega, Madison, U.S.A.) according to the manufacturer's instructions. TNF-α (50 ng/mL) was used as positive control.

### Colonic biopsies

Biopsies for immunohistochemical and qPCR analysis were taken from patients with CD and UC undergoing routine diagnostic colonoscopy. Written informed consent was obtained from all study patients.

### Immunohistochemical and immunofluorescent staining

Immunohistochemical staining of paraffin-embedded tissue of colonic biopsies was performed using standard procedures. Briefly, sections were subjected to heat-induced epitope retrieval solution(pH = 6) and endogenous peroxidase was blocked with 0.3% H_2_O_2_, followed by blocking in 5% normal serum (Dako, Glostrup, Denmark) and avidin and biotin blocking solution (Vector Laboratories, CA, U.S.A.). Primary anti-OSM, antiOSMR, anti-LIFR and anti-gp130 antibodies (Santa Cruz Biotechnology, Santa Cruz, CA, U.S.A.) was applied overnight at 4°C, and detected using a biotinylated secondary antibody together with streptavidin-HRP (all from Dako, Glostrup, Denmark). For immunofluorescent staining, HCT116 cells were fixed for 1 minute in methanol∶glacial acetic acid (1∶1), washed and rehydrated in PBS for at least 30 minutes. Blocking was done for 1 hour at room temperature (RT) in 5% normal serum, followed by incubation with the respective primary antibodies or isotype controls overnight at 4°C. The next day, immunofluorescent staining was achieved by incubation with the appropriate secondary antibody (AlexaFluor488- or AlexaFluor546-conjugated, Life Technologies, Darmstadt, Germany) for 30 minutes at RT. Nuclei were stained with DAPI (Life Technologies, Darmstadt, Germany) and sections were mounted and images acquired on a Zeiss LSM 510 confocal microscope (Zeiss, Jena, Germany).

### Measurement of OSM mRNA levels in colonic biopsies of IBD patients

RNA from colonic biopsies was isolated using ceramic bead-assisted homogenization in a MagNALyser (Roche, Mannheim, Germany). RNA was reverse transcribed and subjected to qPCR. mRNA levels were calculated using a standard-curve which was previously acquired by the measurement of reverse-transcribed serial dilutions of pooled RNA. These arbitrary values were normalized to the median mRNA level of the housekeeping genes *YWHAZ, HPRT1* and *RPL13A*. In cases where no OSM expression was detected, the cycle threshold of the corresponding sample was set to that of the negative control (no reverse transcriptase control).

### Statistical analysis

If not stated otherwise, statistical analyses were performed by using the two-tailed Student's t-test. P values<0.05 were considered as statistically significant. Standard errors of the mean (SEM) were calculated by dividing the standard deviation (SD) by the square root of the number of single data in the respective group.

## Results

### Intestinal epithelial cells express OSM receptors

**Figure 1:**
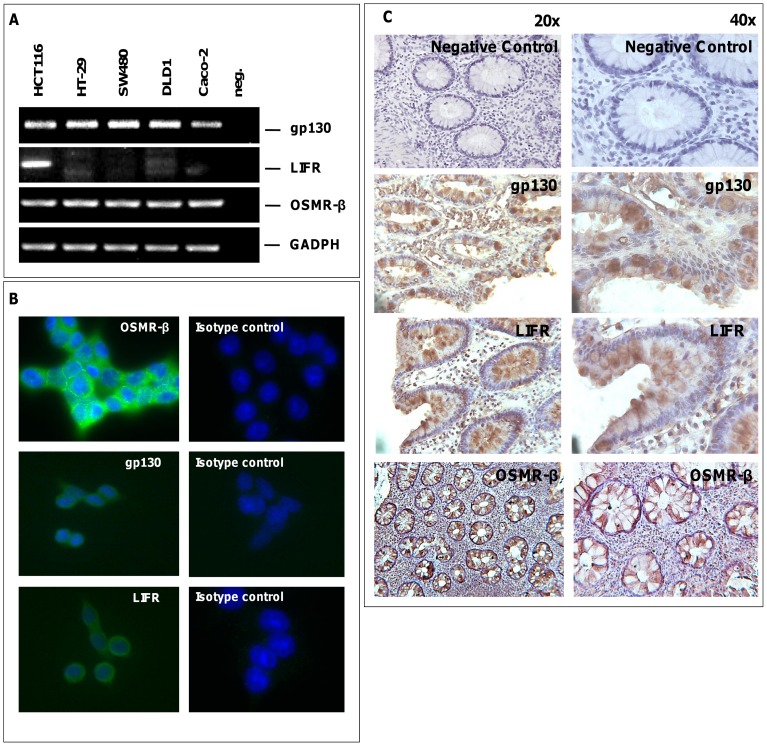
The OSM receptor complexes are expressed in intestinal epithelial cells. (A) mRNA expression of gp130, LIFR and OSMR-β in various intestinal epithelial cell lines, were analyzed by semiquantitative RT-PCR analysis of mRNA derived from cells as indicated. Sterile water served as negative control. (B) Immunofluorescence analysis demonstrated the expression of gp130, LIFR and OSMR-β in HCT116 cells. Nuclei (blue) were stained with DAPI. In the isotype controls (rabbit for LIFR and gp139 and goat for OSMR-β) no specific stainings were detected. (C) Immunhistochemical experiments in colonic biopsies of healthy volunteers, which confirms the expression of the OSM receptors gp130, LIFR and OSMR-β in the human intestine (20× magnification left panel, 40× magnificantion right panel).

To determine if the OSM receptor complexes consisting of gp130 and LIFR (type I) and gp130 and OSMR-β (type II) are expressed in IEC, we analyzed mRNA expression in several human IEC lines (CaCo-2, DLD-1, SW480, HCT116, HT-29). RT-PCR analysis demonstrated gp130 and OSMR-β mRNA expression in all cell lines tested, whereas LIFR was only expressed in HCT116 cells. Accordingly, qPCR analyses confirmed that HCT116 cells express all receptors at a higher level compared to other IEC (Fig. 1A and Fig. S1). Immunofluorescence analysis confirmed OSMR-β, gp130 and LIFR protein expression in HCT116 cells, which were therefore used in the following experiments (Fig. 1B). Moreover, we performed immunhistochemical stainings in colonic biopsies of healthy volunteers which confirmed the expression of the OSM receptors gp130, LIFR and OSMR-β in the human intestine (Fig. 1C).

### OSM activates the MAP kinases ERK-1/2, SAPK/JNK-1/2, and the PI3 kinase Akt and induces phosphorylation of the STAT1 and STAT3 transcription factors in intestinal epithelial cells

**Figure 2:**
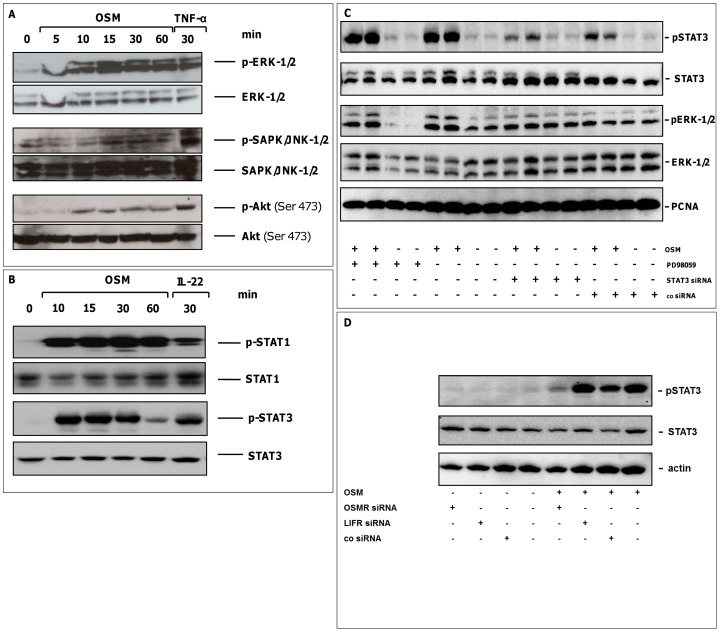
OSM activates ERK-1/2, SAPK/JNK-1/2 MAP kinases, the PI3-kinase Akt and STAT1/3 in HCT116 cells and STAT3 activation is dependent of the LIFR. (A) Stimulation with OSM (100 ng/mL) resulted in increased phosphorylation of ERK-1/2, SAPK/JNK-1/2 kinases and induced PI3 kinase-dependent Akt phosphorylation. One representative experiment (n = 3) is shown. TNF-α (50 ng/mL) served as positive control. (B) Activation and expression of phospho-STATs and protein loading of the respective STAT proteins were assessed by immunoblotting. STAT1 and STAT3 phosphorylation was detected with a maximum at 10–30 min after stimulation with OSM (100 ng/mL). One representative experiment (n = 3) is shown. IL-22 (100 ng/mL), for which we previously demonstrated STAT1 and STAT3 activation in IEC [44], served as positive control. (C) MEK inhibition did not alter OSM-mediated STAT3 upregulation. Similarly, STAT3 inhibition had no significant effect on ERK-1/2 regulation. PCNA served as house keeping control. (D) Knockdown of OSMR, but not LIFR in HCT116 cells resulted in decrease of STAT3-phosphorylation after OSM stimulation compared to cells treated with control siRNA (co siRNA). One representative experiment (n = 3) is shown.

To analyze if the IEC-expressed OSM receptor complexes are functional, we investigated MAP kinase and STAT signaling upon OSM stimulation in IEC. OSM induced phosphorylation of ERK-1/2 and SAPK/JNK-1/2 MAPK (Fig. 2A), whereas total ERK-1/2 and SAPK/JNK-1/2 levels remained unchanged. In addition, we detected an increased phosphorylation of Akt after OSM stimulation (Fig. 2A). Since recent studies have demonstrated STAT activation by OSM in several cell types, we analyzed the influence of OSM on phosphorylation levels of STAT1 and STAT3 in IEC. Tyrosine phosphorylation of STAT1 was increased after stimulation with OSM, as well as tyrosine phosphorylation of STAT3 with a transient and time-dependent maximum phosphorylation level after 10–30 minutes (Fig. 2B).

To determine if MEK inhibition modulates STAT3 activation and *vice versa*, we performed western blots experiments analyzing pERK-1/2 and pSTAT3 after 60 min of OSM stimulation with and without pretreatment with siRNA against STAT3 and the MEK inhibitor PD98059. We demonstrated that there is no crosstalk between MEK and STAT signaling, since MEK inhibition did not alter or diminish OSM-mediated STAT3 upregulation. Similarly, STAT3 inhibition had no significant effect on ERK-1/2 regulation. Significant knockdown of STAT3 using STAT3 siRNA and ERK1/2 using PD98059 is also shown in this experiment (Fig. 2C).

### STAT3 phosphorylation mediated by OSM is dependent on OSMR

To determine which receptor subunit is crucial for STAT3 phosphorylation induced by OSM, we performed western blot experiments with specific siRNA knockdown of LIFR and OSMR in HCT116 cells which express both receptors. We demonstrated that knockdown of the OSMR resulted in a decrease of STAT3 phosphorylation after OSM stimulation compared to cells treated with control siRNA, while pretreatment with LIFR siRNA had no inhibitory effect on STAT3 phosphorylation (Fig. 2D).

### Genes involved in immunity, defense and apoptosis are upregulated upon OSM stimulation of intestinal epithelial cells

Having demonstrated that OSM activates MAP kinases, PI3 kinase and STATs, we next analyzed the impact of OSM on the expression of putative target genes. The OSM-induced gene expression in HCT116 cells was analyzed by RNA microarray experiments. Cells were stimulated for 6 hours with 100 ng/mL OSM, while controls were left unstimulated for the same time interval. A total of 94 genes were significantly regulated by OSM (79 genes up-regulated, 15 down-regulated; adj.-p≤0.05; Table 2). The 20 most strongly up- and downregulated genes for OSM are shown in tables 3 and 4, respectively.

**Table 2:** Number of up- and down-regulated genes after 6 hours with different stringency criteria.

	adj.-p≤0.01			adj.-p≤0.05		
	FC≥2			FC≥2		
	up	down	Σ	up	down	Σ
OSM vs. untreated	50	9	59	79	15	94

The left group represents hits from the initial screening with uncorrected p-value of ≤0.01 vs. untreated cells. The right group depicts the number of regulated genes after the p-value was adjusted for multiple testing. FC = fold-change.

**Table 3:** Top 20 genes whose expression was most strongly induced (p<0.01) by OSM in HCT116 cells after 6 hours of stimulation vs. unstimulated HCT116 cells.

Gene ID	Gene symbol	Description	OSM treatment fold increase
NM_002974	SERPINB4	Homo sapiens serpin peptidase inhibitor, clade B (ovalbumin), member 4 (SERPINB4), mRNA [NM_002974]	39.43
NM_006919	SERPINB3	Homo sapiens serpin peptidase inhibitor. clade B (ovalbumin). member 3 (SERPINB3). mRNA [NM_006919]	36.31
NM_152321	ERP27	Homo sapiens chromosome 12 open reading frame 46 (C12orf46). mRNA [NM_152321]	10.42
NM_001085	SERPINA3	Homo sapiens serpin peptidase inhibitor. clade A (alpha-1 antiproteinase. antitrypsin). member 3 (SERPINA3). mRNA [NM_001085]	9.16
NM_030641	APOL6	Homo sapiens apolipoprotein L. 6 (APOL6). mRNA [NM_030641]	6.65
NM_145641	APOL3	Homo sapiens apolipoprotein L. 3 (APOL3). transcript variant beta/a. mRNA [NM_145641]	5.63
NM_002241	KCNJ10	Homo sapiens potassium inwardly-rectifying channel. subfamily J. member 10 (KCNJ10). mRNA [NM_002241]	5.25
NM_003955	SOCS3	Homo sapiens suppressor of cytokine signaling 3 (SOCS3). mRNA [NM_003955]	5.09
NM_003955	SOCS3	Homo sapiens suppressor of cytokine signaling 3 (SOCS3). mRNA [NM_003955]	5.02
NM_005178	BCL3	Homo sapiens B-cell CLL/lymphoma 3 (BCL3). mRNA [NM_005178]	4.54
THC2664989	THC2664989	Q40J89_EHRCH (Q40J89) Cation efflux protein. partial (6%) [THC2733597]	4.07
NM_031458	PARP9	Homo sapiens poly (ADP-ribose) polymerase family. member 9 (PARP9). mRNA [NM_031458]	4.05
NM_000246	CIITA	Homo sapiens class II. major histocompatibility complex. transactivator (CIITA). mRNA [NM_000246]	3.92
NM_000204	CFI	Homo sapiens complement factor I (CFI). mRNA [NM_000204]	3.64
NM_001712	CEACAM1	Homo sapiens carcinoembryonic antigen-related cell adhesion molecule 1 (biliary glycoprotein) (CEACAM1). transcript variant 1. mRNA [NM_001712]	3.58
NM_030641	APOL6	Homo sapiens apolipoprotein L. 6 (APOL6). mRNA [NM_030641]	3.38
NM_032945	TNFRSF6B	Homo sapiens tumor necrosis factor receptor superfamily. member 6b. decoy (TNFRSF6B). transcript variant M68C. mRNA [NM_032945]	3.37
NM_144586	LYPD1	Homo sapiens LY6/PLAUR domain containing 1 (LYPD1). transcript variant 1. mRNA [NM_144586]	3.13
NM_032206	NLRC5	Homo sapiens NLR family. CARD domain containing 5 (NLRC5). mRNA [NM_032206]	3.13
NM_138287	DTX3L	Homo sapiens deltex 3-like (Drosophila) (DTX3L). mRNA [NM_138287]	3.04

**Table 4:** Top 20 genes whose expression was most strongly repressed (p<0.01) by OSM in HCT116 cells after 6 hours of stimulation vs. unstimulated HCT116 cells.

Gene ID	Gene symbol	Description	OSM treatment fold decrease
AF086547	AF086547	Homo sapiens full length insert cDNA clone ZE12B03. [AF086547]	2.99
ENST00000315707	ENST00000315707	Uncharacterized protein C17orf44. [Source:Uniprot/SWISSPROT;Acc:Q8NAT9] [ENST00000315707]	2.79
AK092638	AK092638	Homo sapiens cDNA FLJ35319 fis, clone PROST2011577. [AK092638]	2.37
A_23_P90470	A_23_P90470		2.18
NM_024320	ATAD4	Homo sapiens ATPase family, AAA domain containing 4 (ATAD4), mRNA [NM_024320]	2.17
NM_145176	SLC2A12	Homo sapiens solute carrier family 2 (facilitated glucose transporter), member 12 (SLC2A12), mRNA [NM_145176]	2.16
NM_005502	ABCA1	Homo sapiens ATP-binding cassette, sub-family A (ABC1), member 1 (ABCA1), mRNA [NM_005502]	2.15
NM_004354	CCNG2	Homo sapiens cyclin G2 (CCNG2), mRNA [NM_004354]	2.14
NM_000499	CYP1A1	Homo sapiens cytochrome P450, family 1, subfamily A, polypeptide 1 (CYP1A1), mRNA [NM_000499]	2.09
NM_152500	CCDC17	Homo sapiens coiled-coil domain containing 17 (CCDC17), mRNA [NM_152500]	2.08
NM_014417	BBC3	Homo sapiens BCL2 binding component 3 (BBC3), mRNA [NM_014417]	2.07
NM_001080474	FLJ43987	Homo sapiens similar to RIKEN cDNA 4930433I11 gene (FLJ43987), mRNA [NM_001080474]	2.05
NM_014417	BBC3	Homo sapiens BCL2 binding component 3 (BBC3), mRNA [NM_014417]	2.03
AK091132	AK091132	Homo sapiens cDNA FLJ33813 fis, clone CTONG2002744. [AK091132]	2.01
NM_006813	PNRC1	Homo sapiens proline-rich nuclear receptor coactivator 1 (PNRC1), mRNA [NM_006813]	2.01
NM_001008540	CXCR4	Homo sapiens chemokine (C-X-C motif) receptor 4 (CXCR4), transcript variant 1, mRNA [NM_001008540]	1.95
BC039664	LOC400604	Homo sapiens hypothetical gene supported by BC039664, mRNA (cDNA clone IMAGE:2901155). [BC039664]	1.94
THC2648781	THC2648781	Q7SCZ7_NEUCR (Q7SCZ7) Predicted protein, partial (5%) [THC2648781]	1.94
AL834280	AL834280		1.89
NM_176891	IFNE1	Homo sapiens interferon epsilon 1 (IFNE1), mRNA [NM_176891]	1.86

**Figure 3:**
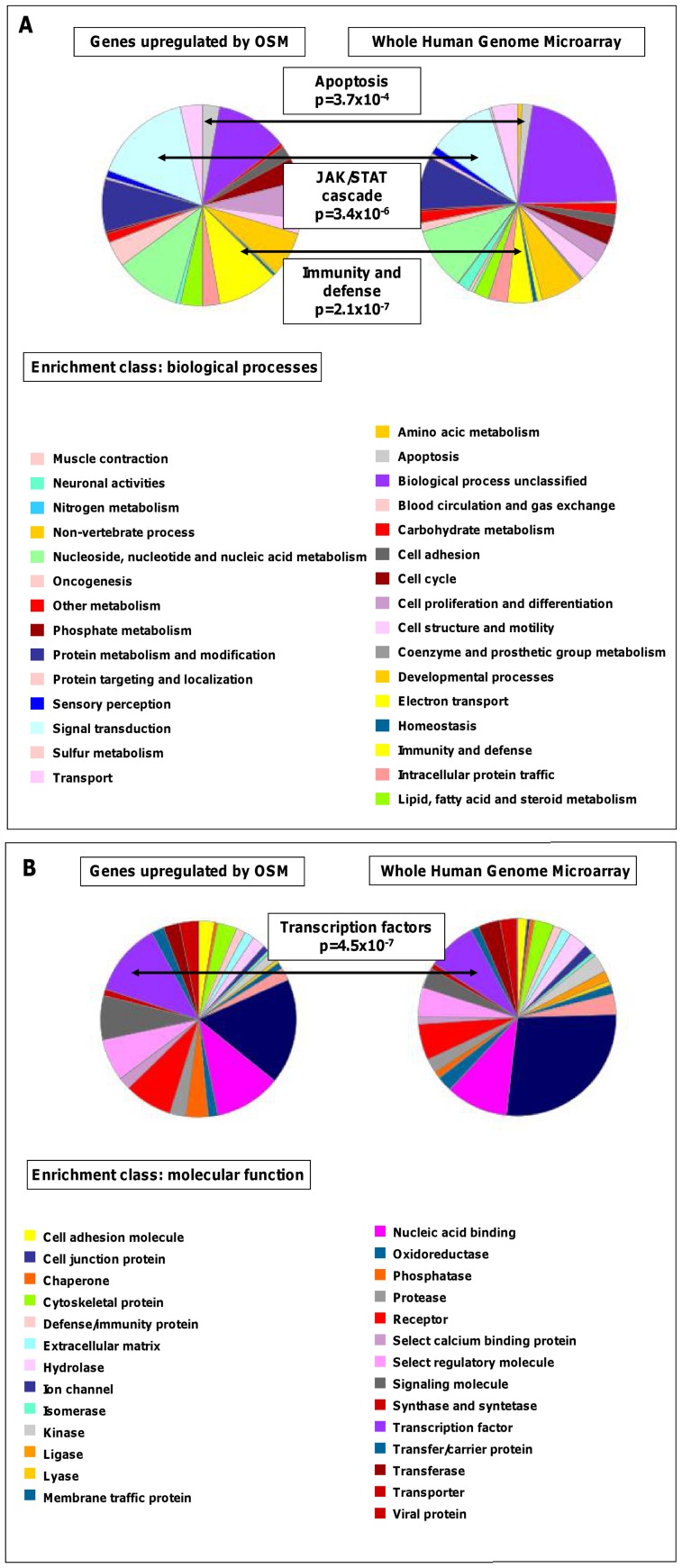
Functional categorization of OSM-induced gene expression. In all classifications, p-values<10^−5^ comparing OSM-induced genes vs. the distribution of all genes on the microarray chip were considered as significant enrichment. Main classification groups are depicted in bold letters. (A) Following OSM stimulation, genes of the biological processes, “immunity and defense” (p = 2.1×10^−7^), “apoptosis” (p = 3.7×10^−4^) and “JAK/STAT cascade” (p = 3.4×10^−6^) were significantly enriched after OSM stimulation. (B) Within the enrichment class molecular functions, “transcription factors” (p = 4.5×10^−7^) were the most strongly upregulated gene group following OSM stimulation. In the legends, the gene classes are listed in a clock-wise order, starting at the “12 o'clock” position.

The differentially regulated genes were analyzed for affiliation to biological processes, molecular functions and pathways using the Panther classification [14]. Enrichment of these functional classes compared to the distribution represented on the Agilent Whole Human Genome microarray was analyzed (Table 5). Within the set of up-regulated genes of biological processes, the classes “immunity and defense” (p = 2.1×10^−7^) and “apoptosis” (p = 3.7×10^−4^) were most significantly enriched (Fig. 3A). In addition, JAK-STAT cascade, a subclass of the biological process class of “signal transduction” was significantly enriched. Among the up-regulated genes of class “other transcription factor”, a subclass of the molecular function class of “transcription factor”, was significantly enriched (Fig. 3B; p = 4.5×10^−7^).

**Table 5:** Functional classification of OSM-induced genes in HCT116 cells after 6 hours of stimulation regarding the categories of biological processes and molecular functions.

**Enrichment class:biological processes**	**induced by OSM (p-value)**
Apoptosis	3.7×10^−4^
JAK-STAT cascade	3.4×10^−6^
Immunity and defense	2.1×10^−7^
Interferon-mediated immunity	4.5×10^−7^
**Enrichment class: molecular functions**	**induced by OSM (p-value)**
Other transcription factor	4.5×10^−7^
Serin protease inhibitor	2.8×10^−5^
**Enrichment class: pathways**	**induced by OSM (p-value)**
Interleukin signaling pathway	7.1×10^−5^

p-values<10^−5^ vs. the distribution of all genes on the microarray chip were considered as significant enrichment.

The genes up-regulated by OSM comprised genes of “immunity, defense and apoptosis”, including serine protease inhibitors (SERPIN)-gene family members (SERPINB4: 39.4-fold, SERPINB3: 36.3-fold, SERPINA3: 9.2-fold; all p<0.01; Table 3).

In addition, SOCS3 (suppressor of cytokine signaling-3), which represents a negative feedback regulator of cytokine signaling, was up-regulated by OSM in microarray (5-fold). Interestingly, colonic tissue samples of patients with Crohn's disease demonstrated increased STAT1 phosphorylation levels and, compared to samples taken from ulcerative colitis patients, increased SOCS3 levels [17]. Another gene upregulated 4-fold by OSM is CIITA (class II, major histocompatibility complex, transactivator), which we recently demonstrated as a target gene of the STAT3-activating cytokine IL-27 [18]. Moreover, mucin 1 (MUC-1) was up-regulated 2.3-fold by OSM, which enhances vascular endothelial growth factor (VEGF) via the Akt signalling pathway [19]. NLRC5, a member of the family of NOD-like receptors and intracellular sentinel proteins, which are implicated in the detection of microbes and danger signals and thereby controlling several key innate immune pathways, was 3.1-fold up-regulated by OSM. This receptor is known to be a negative modulator of inflammatory pathways [20], underlying potential protective effects of OSM in intestinal inflammation. Carcinoembryonic antigen-related cell adhesion molecule 1 (CEACAM1), upregulated 3.6-fold by OSM, is a protein expressed by T cells which functions as a coinhibitory receptor after T cell activation. Deficiency in the expression of CEACAM1 has been described in IBD, might therefore be a novel potential therapeutic target in the treatment of IBD [21], [22]. Two SNPs on chromosomes 20q13 (rs2315008 and rs4809330), which are closely located to the TNFRSF6B gene, which is upregulated 3.4-fold by OSM, have been associated with pediatric-onset of IBD [23].

### STAT proteins (STAT1, STAT2, STAT3, STAT4, STAT5A, STAT5B, STAT6) are upregulated after OSM stimulation in HCT116 cells

We investigated regulation of all STAT proteins (STAT1, STAT2, STAT3, STAT4, STAT5A, STAT5B, STAT6) via qPCR after 60 min OSM stimulation in HCT116 cells (Fig. S2). We found a 2-fold increase of STAT1 and STAT3, 1.5-fold increase of STAT6 vs. unstimulated HCT116 cells, while there was no effect for STAT4. The most significant increase was found for STAT5A, which was up to 16-fold increased vs. unstimulated cells, while there was only a moderate increase (1.6-fold) for STAT5B. Importantly, STAT5 is crucial for cell proliferation, differentiation and apoptosis [24]. This is in line with published data from fibroblast-like synoviocytes isolated from patients with rheumatoid arthritis and stimulated with recombinant OSM, in which OSM increased STAT1, STAT3 and STAT5 expression [25].

### OSM-induced SERPINA3 gene expression is STAT3-dependent

**Figure 4:**
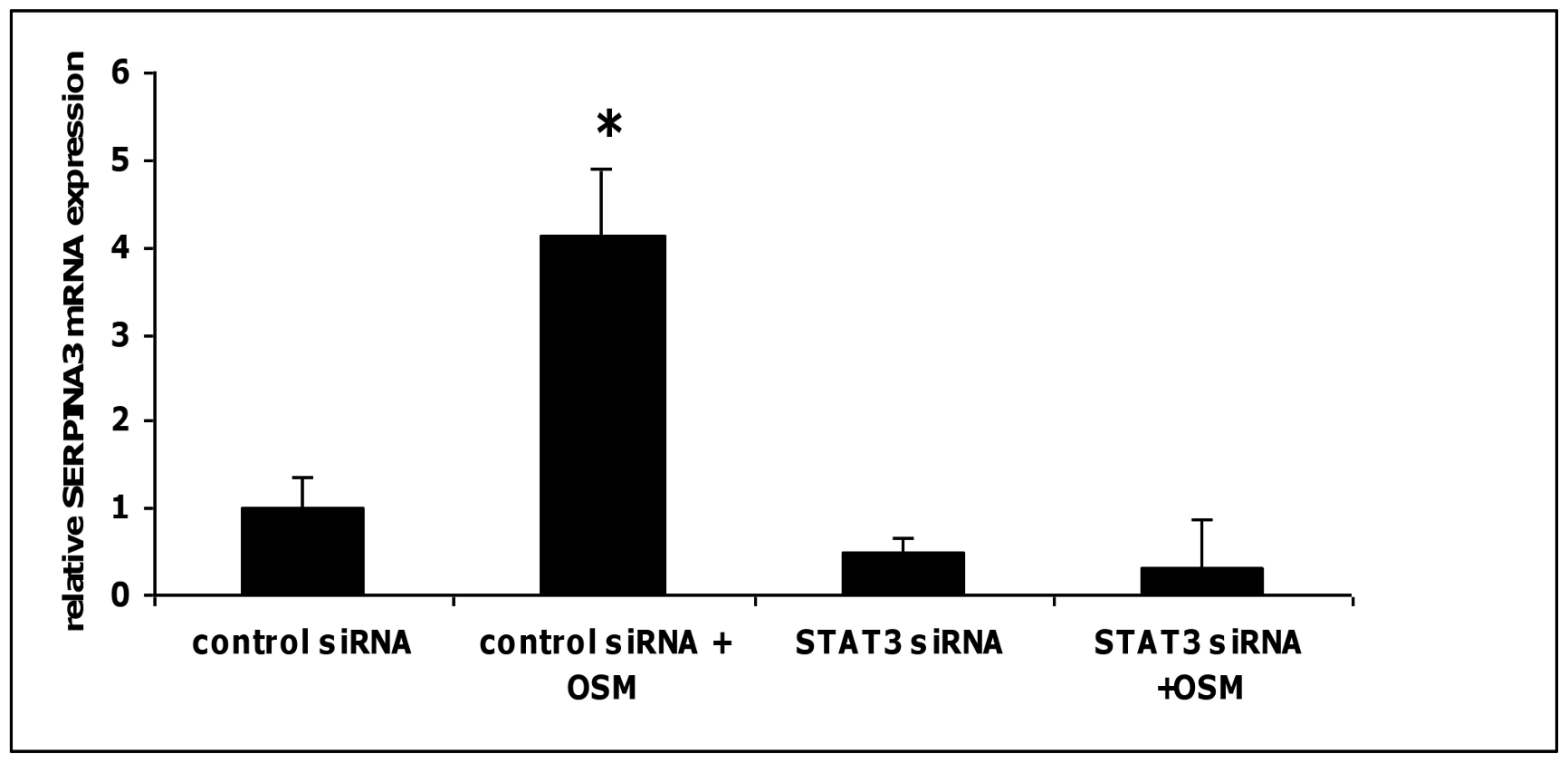
OSM-induced SERPINA3 expression is STAT3-dependent. Stimulation with OSM induced SERPINA3 mRNA (*p = 0.02) and transfection with STAT3 siRNA abrogated OSM-mediated induction of SERPINA3 mRNA; (p = 0.22, OSM-stimulated vs. unstimulated STAT3 siRNA transfected HCT116 cells). SERPINA3 mRNA expression in unstimulated control cells was arbitrarily set to 1.0.

STAT3 is known to be a key transcription factor which mediates OSM signaling in several cell systems. Therefore, we further investigated, if SERPINA3 upregulation is STAT3-dependent, since SERPINA3 has been shown to be a prototypic acute phase molecule up-regulated upon OSM stimulation in hepatoma cells [11] as also demonstrated in IEC in our microarray experiments. Using quantitative PCR analysis, OSM significantly upregulated SERPINA3 mRNA expression (p = 0.02, Student's t-test; OSM-stimulated vs. unstimulated control siRNA transfected HCT116 cells), while siRNA-mediated knock-down of STAT3 almost completely abolished up-regulation of SERPINA3 mRNA (p = 0.22, Student's t-test; OSM-stimulated vs. unstimulated STAT3 siRNA transfected HCT116 cells) upregulation after stimulation of HCT116 cells with OSM (Fig. 4).

### OSM promotes intestinal epithelial cell proliferation and wound healing *via* STAT3 and MEK1 kinase signaling pathways

Previous studies have demonstrated that OSM and SERPINA3 induce migration of keratinocytes *in vitro* [26] and skin repair *in vivo* [27], respectively, which implicates an important role of OSM in cell reconstitution. Since activation of STAT3, ERK1/2 and Akt is associated with cell proliferation [21], [22], and microarray analysis revealed up-regulated of genes involved in proliferation and apoptosis by OSM, we next analyzed if OSM promotes wound healing in intestinal epithelial cells.

**Figure 5:**
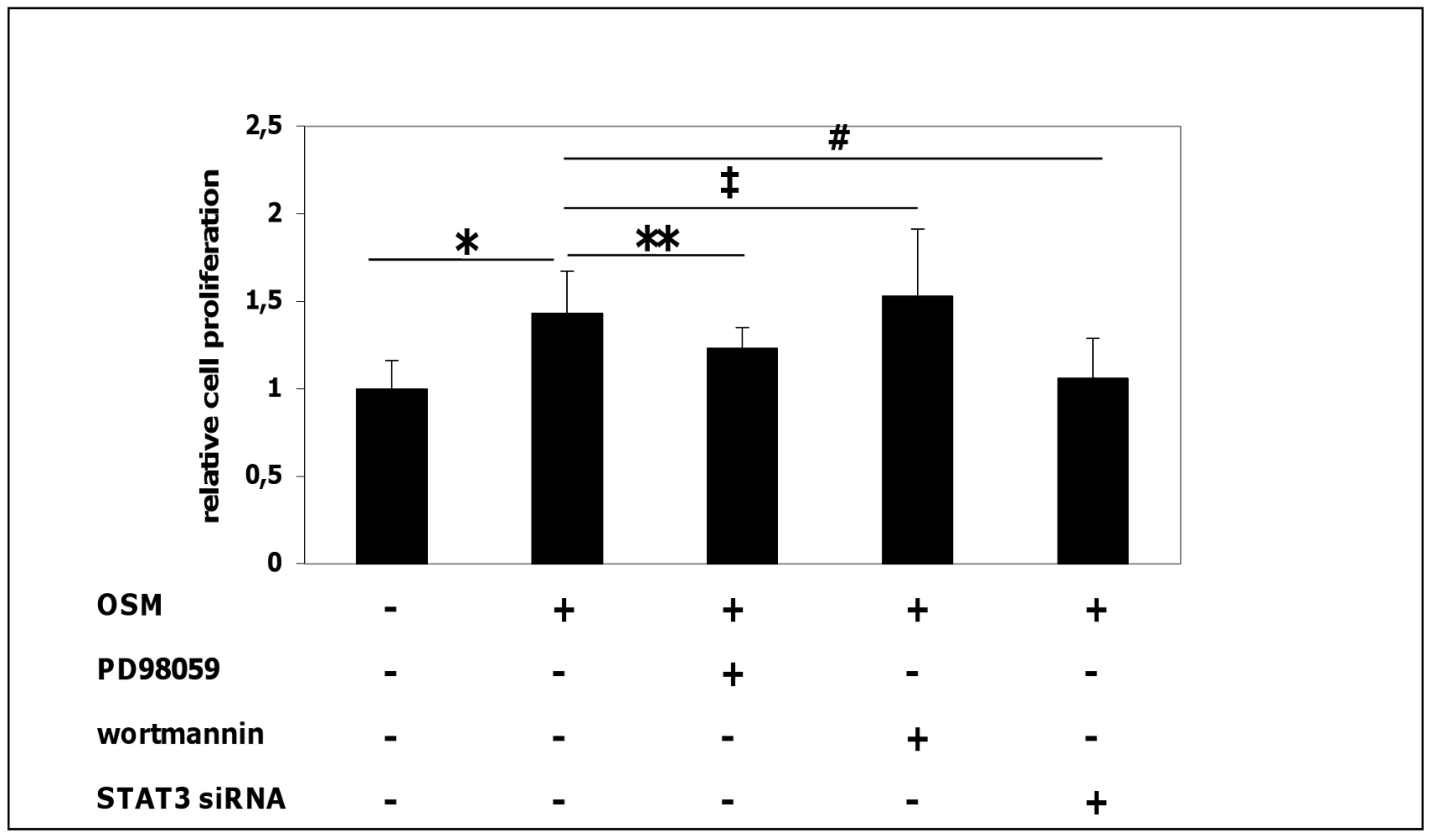
OSM induces cell proliferation in HCT116 cells which is mediated by MEK-1- and STAT3 signaling. After stimulation with 100/mL OSM for 48 hours, cell proliferation was significantly higher in OSM-treated cells in comparison to unstimulated cells as determined by WST-1 assay; * p = 0.02 vs. control. Pretreatment with the ERK-1/2-inhibitor PD98059 and transfection with STAT3 siRNA reduced OSM-mediated cell proliferation, while there was no effect by the PI3 kinase inhibitor wortmannin; ** p = 0.01, ^#^ p = 0.006, **^‡^** p = 0.57 vs. OSM-stimulated. In all experiments, proliferation in unstimulated control cells was arbitrarily set to 1.0.

At 100 ng/mL, OSM significantly increased cell proliferation of HCT116 cells (p = 0.02) measured by WST-1 assay. The OSM-induced cell proliferation was not impaired by the pretreatment with the Akt inhibitor wortmannin (p = 0.57 compared to cells stimulated with OSM only, Fig. 5). In contrast, inhibition of the ERK pathway using the MEK-1 inhibitor PD98059 decreased OSM-mediated cell proliferation (Fig. 5, p = 0.01 compared to OSM stimulated cells). Since expression of the anti-apoptotic protein SERPINA3 upon OSM stimulation was significantly reduced by blocking STAT3, we further investigated if HCT116 cells proliferation is also STAT3-dependent. We could demonstrate, that siRNA-mediated STAT3 knock-down significantly reduced the OSM-induced proliferative effect (Fig. 5, p = 0.006 compared to OSM stimulated cells). Taken together, these finding suggest that OSM-induced cell proliferation is MEK-1 kinase- and STAT3-dependent.

**Figure 6:**
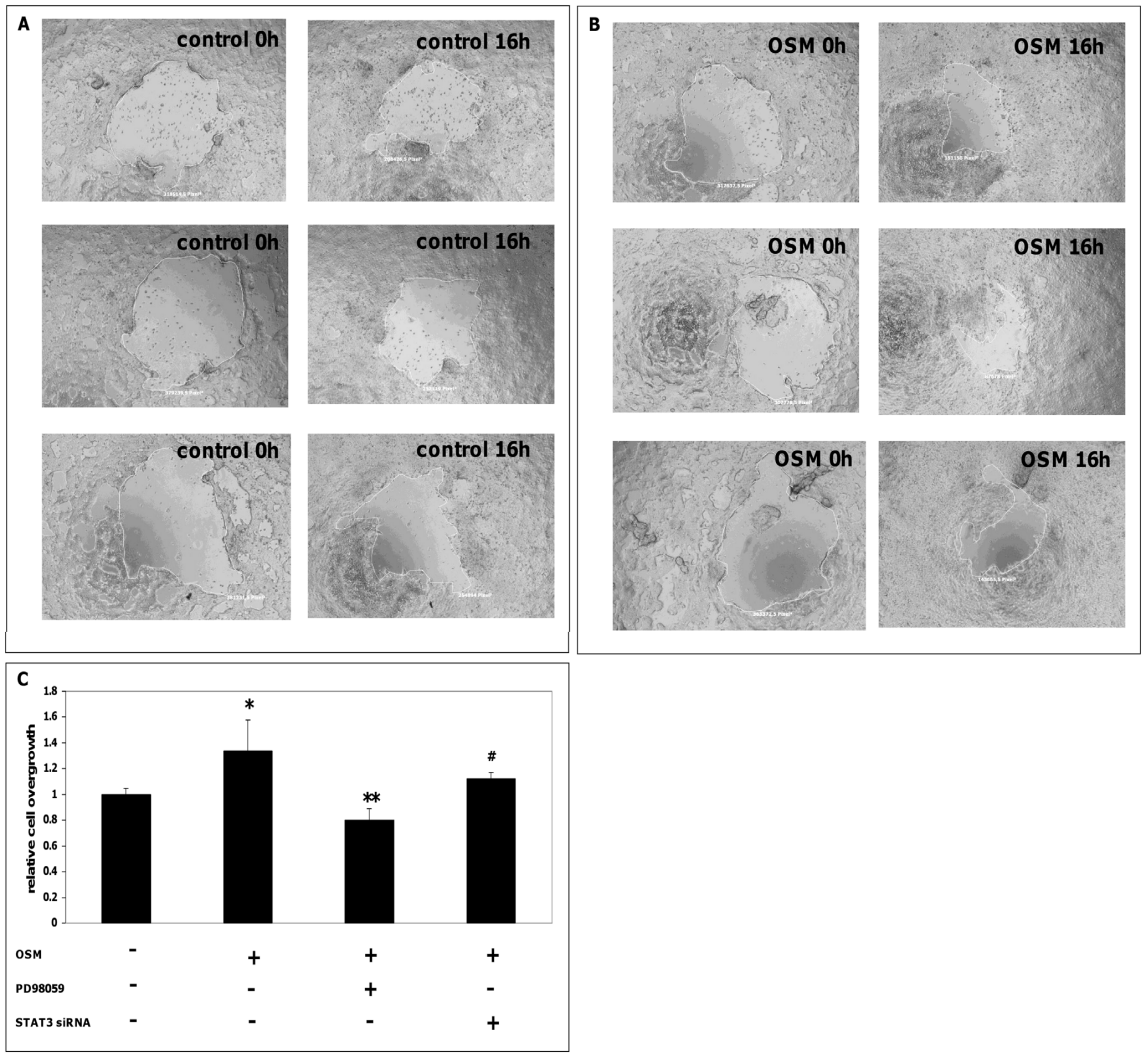
OSM-mediated wound healing in HCT116 cells is MEK-1- and STAT3-dependent. Wounding assays were performed to analyze cell migration. Presented are representative images of unstimulated HCT116 cells at baseline (left panel) and after 16 hours (right panel) (A) and OSM-stimulated cells at baseline (left panel) and after 16 hours (right panel) (B). (C) OSM (100 ng/mL) induced a significant increase of cell migration (* p = 3.9×10^−5^ vs. unstimulated controls). Preincubation with the MEK-1 inhibitor PD98059 (10 µM) and STAT3 siRNA (5 µM) reduced OSM-induced cell migration in the wounding assay (** p = 1.7×10^−5^ and ^#^ p = 0.01 vs. OSM-stimulated cells). In all experiments, relative cell overgrowth in unstimulated control cells was arbitrarily set to 1.0.

Next, we investigated the effect of OSM on IEC wound healing *in vitro*. In standardized wounding assays, sterile circular wounds (Fig. 6A and 6B) were created in HCT116 cells. Sixteen hours after wounding, the overgrown area was measured. These experiments demonstrated a significant increase of overgrown area after OSM stimulation in this assay (p = 3.9×10^−5^ vs. unstimulated controls, Fig. 6C). Since the proliferation assays demonstrated that OSM-induced proliferation is MEK-1- and STAT3-dependent, we pretreated HCT116 cells with the MEK-1 inhibitor PD98059 which resulted in a significant reduction of OSM-mediated cell migration in the wounding assay (p = 1.7×10^−5^ vs. OSM stimulated, Fig. 6C). Pretreatment with a STAT3 inhibitor reduced OSM-mediated cell migration in a similar manner (p = 0.01 vs. OSM stimulated, Fig. 6C)

### Apoptosis of intestinal epithelial cells is down-regulated by OSM stimulation

**Figure 7:**
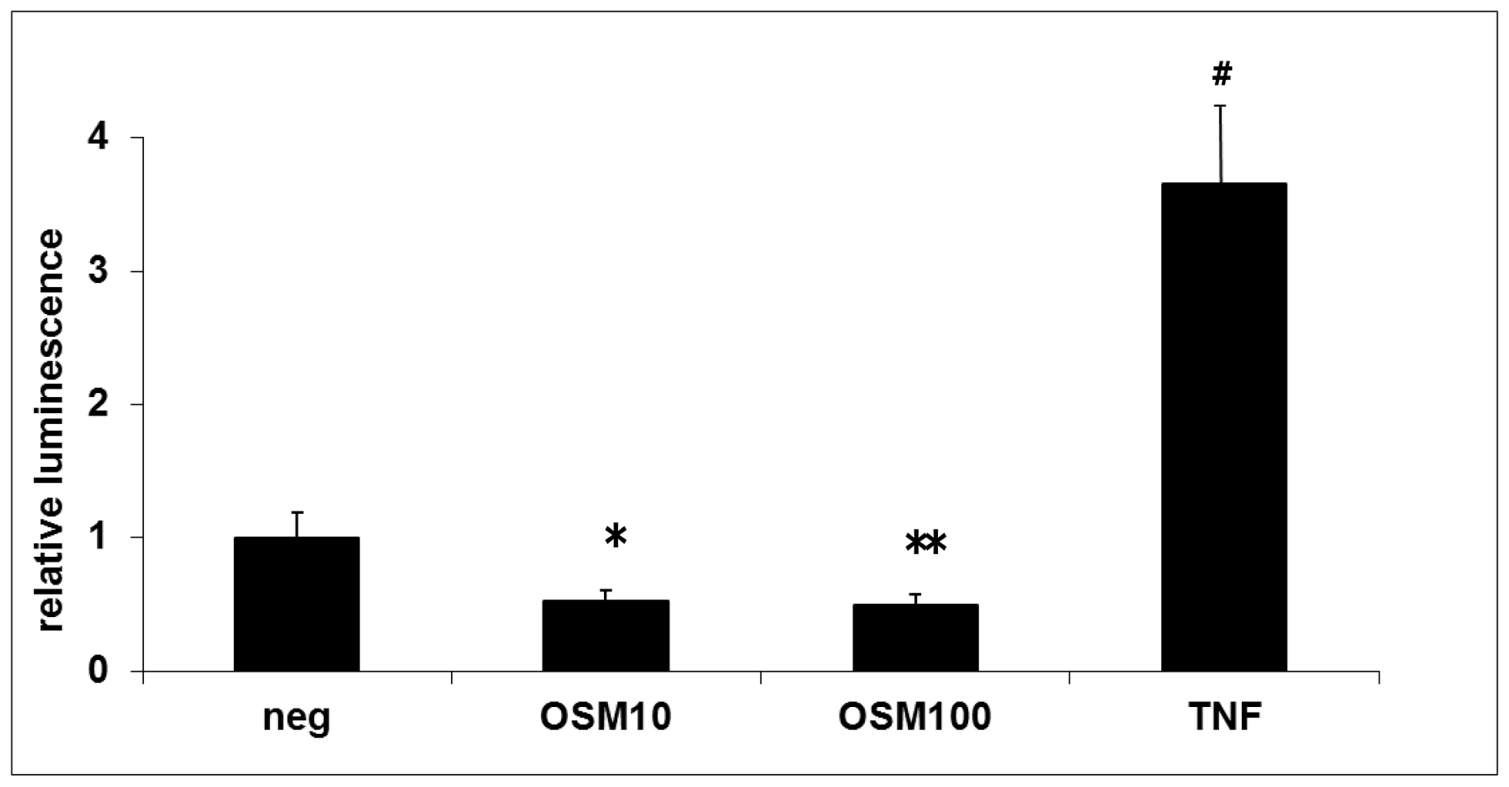
Apoptosis of HCT116 cells is downregulated after OSM stimulation. Treatment with OSM (10 ng/mL and 100 ng/mL) for 6 hours significantly reduced caspase-3/7 activity (* p = 0.03; **p = 0.02, respectively) compared to unstimulated HCT116 cells. TNF-α (50 ng/mL) served as positive control (^#^ p = 0.007 vs. unstimulated HCT116 cells).

In the next step, we analyzed whether the proliferative effects of OSM are the result of decreased apoptosis in IEC, given that STAT3, ERK-1/2 and Akt activation have been linked to anti-apoptotic pathways [28], [29], [30]. Moreover, RNA microarray revealed that anti-apoptotic genes such as SERPINs are strongly up-regulated by OSM. Treatment of HCT116 cells with OSM in the concentrations of 10 ng/mL and 100 ng/mL for 6 hours significantly reduced caspase-3/7 activity compared to unstimulated HCT116 cells (p = 0.03 and p = 0.02, respectively; Figure 7). These findings suggest that the proliferative and “wound healing” effects of OSM in IEC results at least partly from an anti-apoptotic effect of OSM.

### OSM expression is increased in the inflamed colonic mucosa of patients with active inflammatory bowel disease

**Figure 8:**
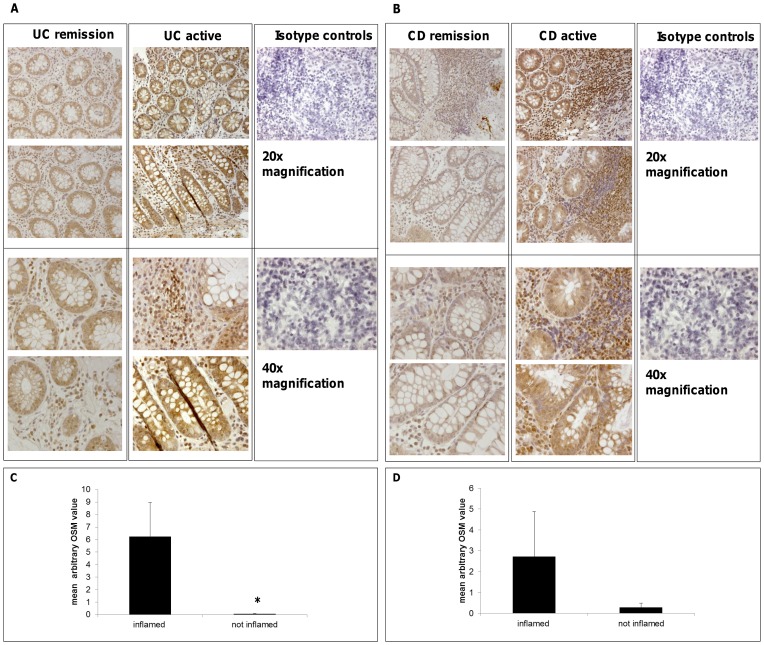
OSM is up-regulated in colonic biopsies of patients with active inflammatory bowel disease. OSM expression in colonic biopsies was higher in inflamed colonic biopsy samples in both active CD and UC patients than in non-inflamed colonic lesions of patients in remission. OSM in inflamed lesions is highly expressed in epithelial cells but also in sub-epithelial laminar propria mononuclear cells. Overall, biopsy specimens of 4 CD and 4 UC patients with active disease and in remission were analyzed. Representative images (20× and 40× magnification) of CD patients in remission and active disease (A) and UC patients in remission and active disease (B) and isotype controls are shown. (C) OSM mRNA levels in inflamed mucosal lesions of 23 patients with CD were significant higher than in non-inflamed lesions (*p = 0.001). (D) OSM mRNA levels were not significant different in inflamed lesions compared to non-inflamed lesions of 10 patients with UC (p = 0.11).

It is assumed that the development of IBD is caused by barrier dysfunction of the intestinal epithelium [31]. Our experiments indicate that OSM promotes wound healing and has proliferative and anti-apoptotic effects on IEC. Thus, we hypothesized that up-regulation of OSM in active IBD might contribute to a stabilization of the intestinal epithelial barrier in a counter-regulatory manner to pro-inflammatory cytokines. We therefore analyzed OSM expression in patients with active and non-active IBD. Criteria for non-active IBD were the absence or minimal signs of inflammatory lesions in the endoscopic and histological analysis of colonic biopsies and also evaluation of clinical activity scores. For CD, the Crohn's disease activity index (CDAI) was calculated and values ≥150 were assigned to active disease. For UC, the colitis activity index (CAI) was calculated and values ≥5 were assigned to active disease. Immunohistochemical analyses for OSM expression in biopsy samples of both patients with active and quiescent Crohn's disease (CD) and ulcerative colitis (UC) showed that OSM staining was more pronounced in inflamed colonic biopsy samples in both active CD and UC patients compared to non-inflamed colonic lesions of IBD patients in remission. OSM expression was found in IEC and more intense staining was found in lamina propria cells (Fig. 8A and 8B). In order to verify increased OSM levels during active IBD, we additionally measured OSM mRNA levels in inflamed vs. non-inflamed mucosa of 33 consecutive IBD patients and found significantly higher OSM levels in inflamed lesions (mean arbitrary OSM value 6.25) compared to non-inflamed lesions (mean arbitrary OSM value 0.07) of patients with CD (p = 0.001), but not in UC (mean arbitrary OSM value inflamed lesions 2.72 vs. non-inflamed lesions 0.28; p = 0.11; Fig. 8C and 8D). CRP values of CD patients correlated with OSM values in inflamed lesions (r = 0.66; Fig. S3), which indicate OSM as a valuable marker of inflammation in CD. Demographical and clinical data of all analyzed patients are provided in Tables S1–3 in File S1.

## Discussion

Oncostatin M (OSM), an IL-6 family member demonstrated ambivalent roles during inflammation in previous studies. Pro-inflammatory properties of OSM were reported in the skin, adipose tissue, lung, heart and liver [32], [33], [34], [35], [36]. In contrast, other studies demonstrated anti-inflammatory effects of OSM, e.g. through up-regulation of antiproteases like α1-antitrypsin in lung epithelial cells [37]. Another study demonstrated that OSM inhibits LPS-induced TNF-α expression [38], which represents a key inflammatory cytokine in inflammatory bowel disease (IBD) and other chronic inflammatory diseases.

In our study, we investigated OSM receptor expression, OSM-mediated biological functions and gene expression patterns in IEC and OSM expression in IBD for the first time. We demonstrated that the OSM receptor complexes I and II are functionally expressed in IEC. Furthermore, OSM induced phosphorylation of the transcription factors STAT1 and STAT3 in IEC. In addition, OSM activates ERK-1/2 and SAPK/JNK-1/2 MAP kinases in IEC. Two recent studies demonstrated that SAPK/JNK-1/2 is activated in Crohn's disease (CD) [39], [40]. Importantly, the activation of ERK-MAP kinases and Akt has been linked to cell migration [41], [42], and in previous studies, we demonstrated that activation of Akt and STAT proteins by various cytokines modulates IEC proliferation and migration [43], [44], [45], [46], [47]. Similarly, our experiments demonstrated that OSM receptor activation results in increased IEC migration and epithelial wound healing which could be blocked using a MEK-1 kinase inhibitor (PD98059) and by siRNA-mediated STAT3 inhibition. Activation of the STAT3 pathway is common for cytokines which mediate cell migration [48], and in line we could demonstrate a STAT3-dependent mechanism for OSM-induced cell migration. We further demonstrated in siRNA gene knockdown experiments that OSM-induced STAT3 phosphorylation in HCT116 cells is dependent on OSMR. STAT3 seems to be the key mediator of IEC wound healing activated by various cytokines, e.g. IL-22 [49]. Very recently, we could demonstrate that the IL-6 family member IL-27, which also signals via gp130, promotes IEC cell restitution via STAT3 [50]. STAT3 was also shown to control the expression of antimicrobial peptides, such as RegIII and S100A proteins. Of note, during skin inflammation, the antimicrobial peptides S100A7 and beta-defensin 2 are upregulated by OSM [26].

The integrity of the intestinal mucosal surface barrier is disrupted in conditions like IBD, and epithelial restitution (mucosal healing) is an important goal of IBD treatment [51]. As demonstrated in wounding assays in our study, OSM stimulation may promote this epithelial restitution. Although our *in vitro* data demonstrated that OSM promotes the integrity of the intestinal barrier, additional *in vivo* experiments are necessary to clarify the role of OSM in intestinal wound healing. An increased cell proliferation after OSM stimulation has been demonstrated in keratinocytes [26] and also wound healing in diabetic mice *in vivo* [52], suggesting that OSM increases the innate immunity of epithelial tissues such as skin and intestine. Moreover, as demonstrated in an OSMR knockout mouse model, OSM is a key mediator of liver regeneration by preventing hepatocyte apoptosis [53], which we also demonstrated in IEC in our study.

Since activated T cells are major sources of OSM [54] and CD represents a T_H_1/T_H_17-mediated type of intestinal inflammation [55], while ulcerative colitis resembles more a T_H_2 mediated colitis [56], OSM may play a crucial role in both disease entities. Previously, we demonstrated that the cytokines IL-22, IL-26, IL-27 and IL-31 are significantly up-regulated in the inflamed mucosa of patients with CD [44], [50], [57], [58]. In this study we showed that OSM expression is increased in inflamed lesions in patients with active CD compared to non-inflamed lesions and that OSM values in inflamed lesions positively correlates with CRP values in these patients. This up-regulation may suggest a counter-regulatory mechanism by which increased OSM expression contributes to stabilization of the intestinal epithelial barrier. Interestingly, the T_H_2 cytokine IL-31 shares the receptor subunit OSMR-β with OSM and we demonstrated, that the two receptor subunits gp130 and OSMR are also expressed at high levels in IEC and are increased in inflamed intestinal tissue [50], [58].

Our microarray analysis of OSM-stimulated vs. unstimulated HCT116 cells revealed activation of genes involved in immunity and defense, interferon-mediated immunity and the JAK-STAT cascade. The most strongly up-regulated genes were SERPINS, which belong to the family of serin peptidase inhibitors with antiprotease activities, most of them serine and cysteine proteases. SERPINB4, SERPINB3 and SERPINA3 were the genes with the strongest up-regulation, also verified in qPCR. Recent data demonstrated that overexpression of SERPINB4 in tumor cells inhibited recombinant granzyme M-induced as well as NK cell-mediated cell death [59]. Accordingly, OSM-induced SERPIN up-regulation may contribute to the anti-apoptotic and proliferative effects of OSM found in our experiments. However, during colitis many proapoptotic cytokines like TNF-α are likely to be present, which might interfere with OSM-mediated effects on apoptosis *in vivo*. It has been shown, that SERPINB3 significantly attenuates apoptosis by contrasting cytochrome c release from the mitochondria and by antichemotactic effects for NK cells [60]. Also, SERPINB3, which is over-expressed in human hepatocellular carcinoma [61], has been shown to induce apoptosis resistance, epithelial-to-mesenchymal transition and increasing cellular invasion [62]. Of note, it is speculated that deregulation of apoptosis plays a major role in autoimmunity characterized by the disability to terminate the immune response. SERPINA3 (α-1 antichymotrypsin) is a secreted acute phase protein strongly associated with numerous inflammatory diseases. Its up-regulation by OSM has formerly been demonstrated in HepG2 cells and rat primary hepatocytes [11]. A recent study showed that SERPINA3 is strongly up-regulated by dexamethasone and the proinflammatory cytokine TNF-α in lung cells *in vitro* and in both lung and liver tissues *in vivo* when C57BL/6 mice were treated with dexamethasone and TNF-α [63]. Moreover, a pivotal role of SERPINA3 has been recently described in skin repair [27]. For SERPINB1, another member of the SERPIN family, which was up-regulated 1.8-fold by OSM in the microarray, a recent study demonstrated protective effects for SERPINB1 in colonic epithelial cells and up-regulation in inflamed colonic tissue of UC patients [64].

In summary, we demonstrated that IEC express functional OSM receptors. Binding of OSM to the OSM receptor leads to phosphorylation of STAT1/3, Akt, and the MAP kinases ERK-1/2 and SAPK-JNK-1/2 in IEC. In microarray experiments, OSM strongly induced genes involved in immunity and defense, interferon-mediated immunity and the JAK-STAT cascade. The most up-regulated genes were SERPINS, which belong to the family of serin peptidase inhibitors which have anti-apoptotic properties. OSM also increased MEK1- and STAT3-dependent IEC proliferation and wound healing. OSM may play therefore a pivotal role in autoimmune processes and gut inflammation potentially mediated via STAT3-dependent up-regulation of SERPINS, which influence apoptosis and therefore cell restitution in inflammatory conditions. The expression of OSM is up-regulated in inflamed colonic lesions in patients with active IBD which may indicate a counter-regulatory role of OSM by stabilizing the disrupted intestinal epithelial barrier. Further *in vivo* mouse studies analyzing functional/conditional knockout of OSM in inflammatory bowel models are warranted to elucidate the exact role of OSM in intestinal inflammation and the potential of the OSM system as a drug target in IBD.

## Supporting Information

Figure S1 **Relative mRNA expression of the OSM receptor units gp130, LIFR and OSMR-β in intestinal epithelial cell lines analyzed by quantitative PCR.** Sterile water served as negative control.
(TIF)

Figure S2 **Relative mRNA expression of STAT 1–6 after stimulation with 100 ng/mL OSM.**
(TIF)

Figure S3 **Correlation between CRP values (mg/dL) and arbitrary OSM values in inflamed lesions of patients with Crohn's disease (r = 0.66).**
(TIF)

File S1 **File S1 includes the following: Table S1**. Demographics of IBD biopsy patients (IHC). **Table S2**. Demographics of Crohn's patients (qPCR). **Table S3**. Demographics of ulcerative colitis patients (qPCR).
(DOCX)
