# Supplementary figures and images for: Oncostatin M Mediates STAT3-Dependent Intestinal Epithelial Restitution via Increased Cell Proliferation, Decreased Apoptosis and Upregulation of SERPIN Family Members
Source: PLoS One. 2014 Apr 7;9(4):e93498. doi: 10.1371/journal.pone.0093498 (PMC3977870; doi:10.1371/journal.pone.0093498)

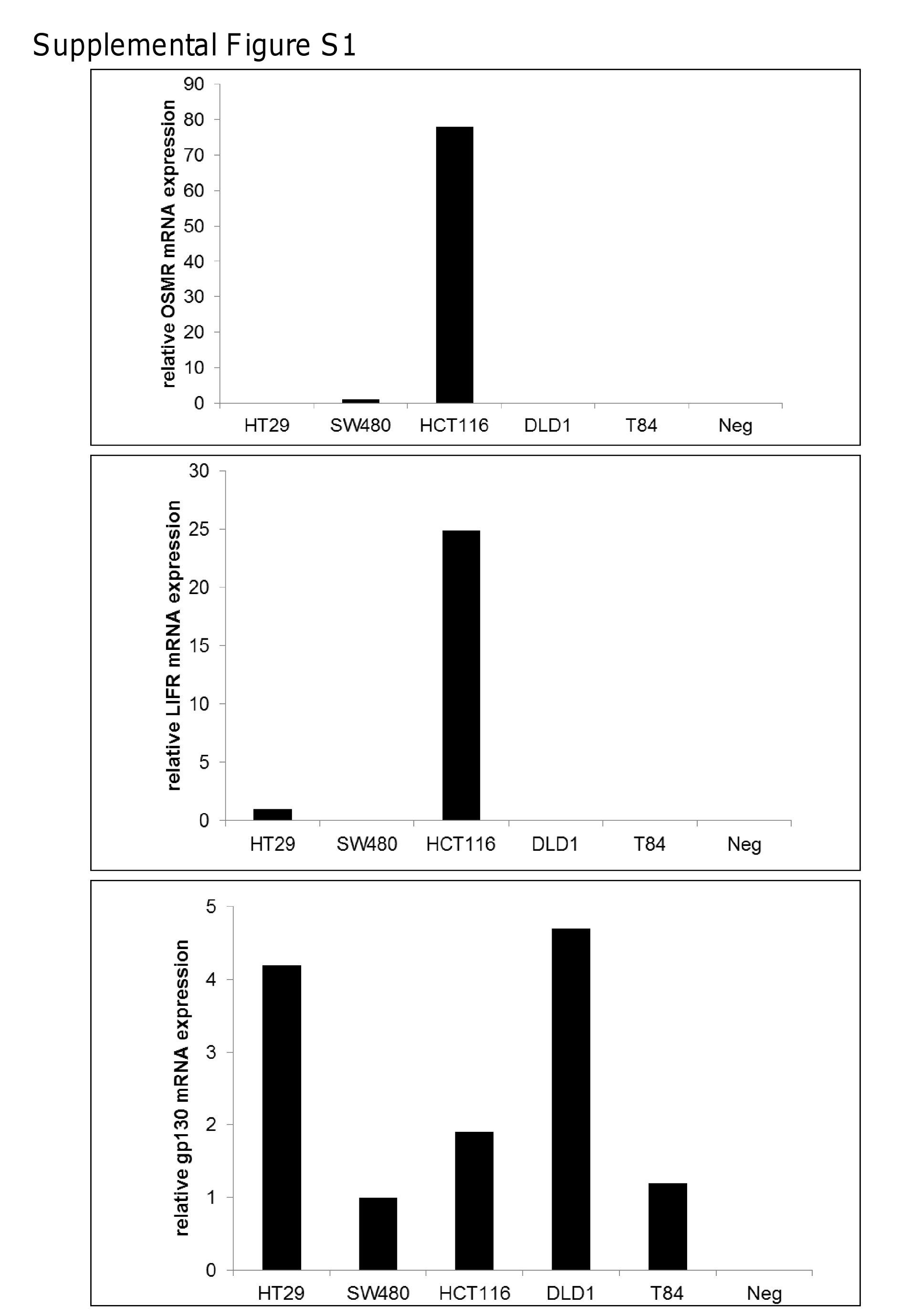

Supplement: Figure S1 — Relative mRNA expression of the OSM receptor units gp130, LIFR and OSMR-β in intestinal epithelial cell lines analyzed by quantitative PCR. Sterile water served as negative control. (TIF) [file pone.0093498.s001.tif]

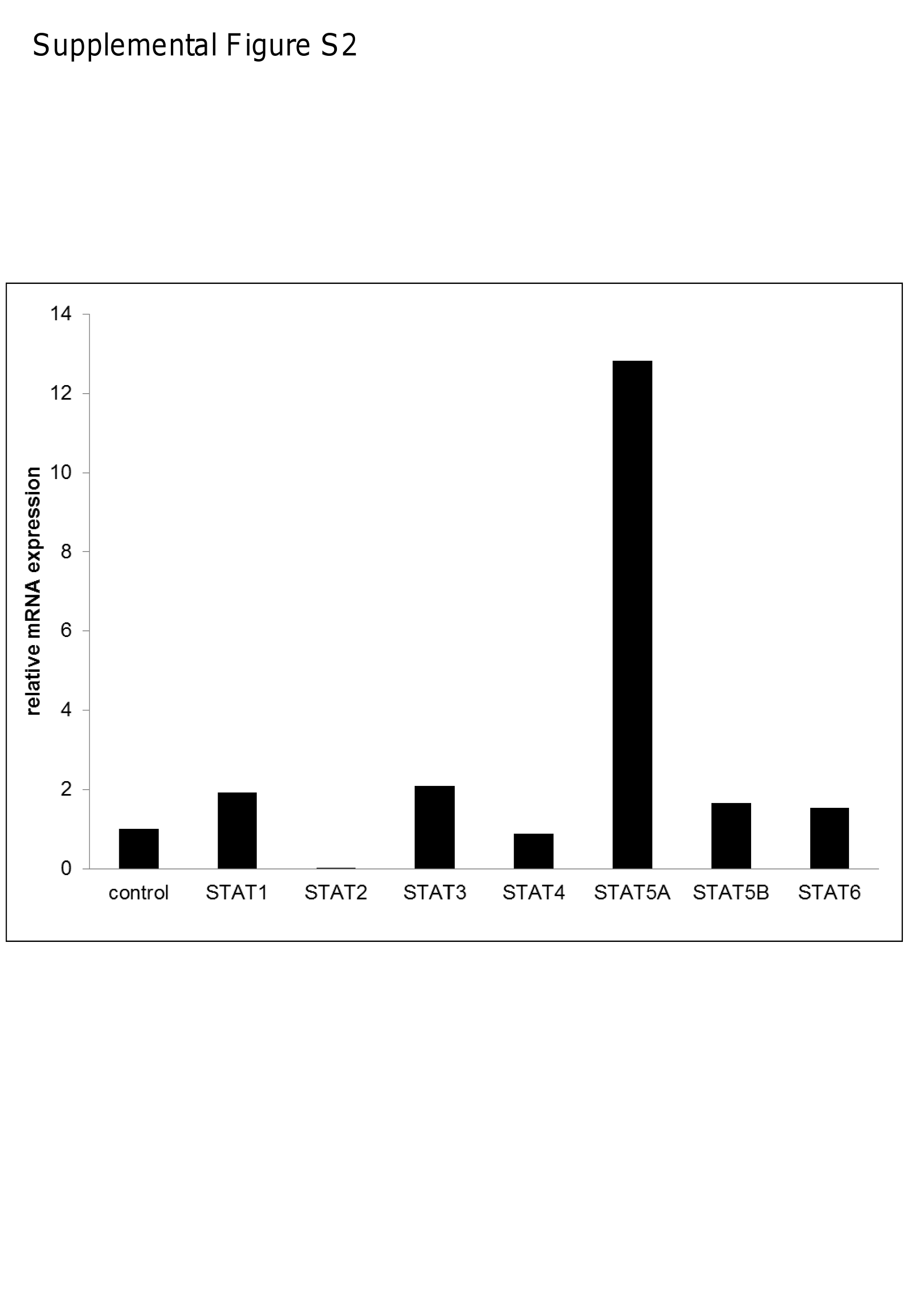

Supplement: Figure S2 — Relative mRNA expression of STAT 1–6 after stimulation with 100 ng/mL OSM. (TIF) [file pone.0093498.s002.tif]

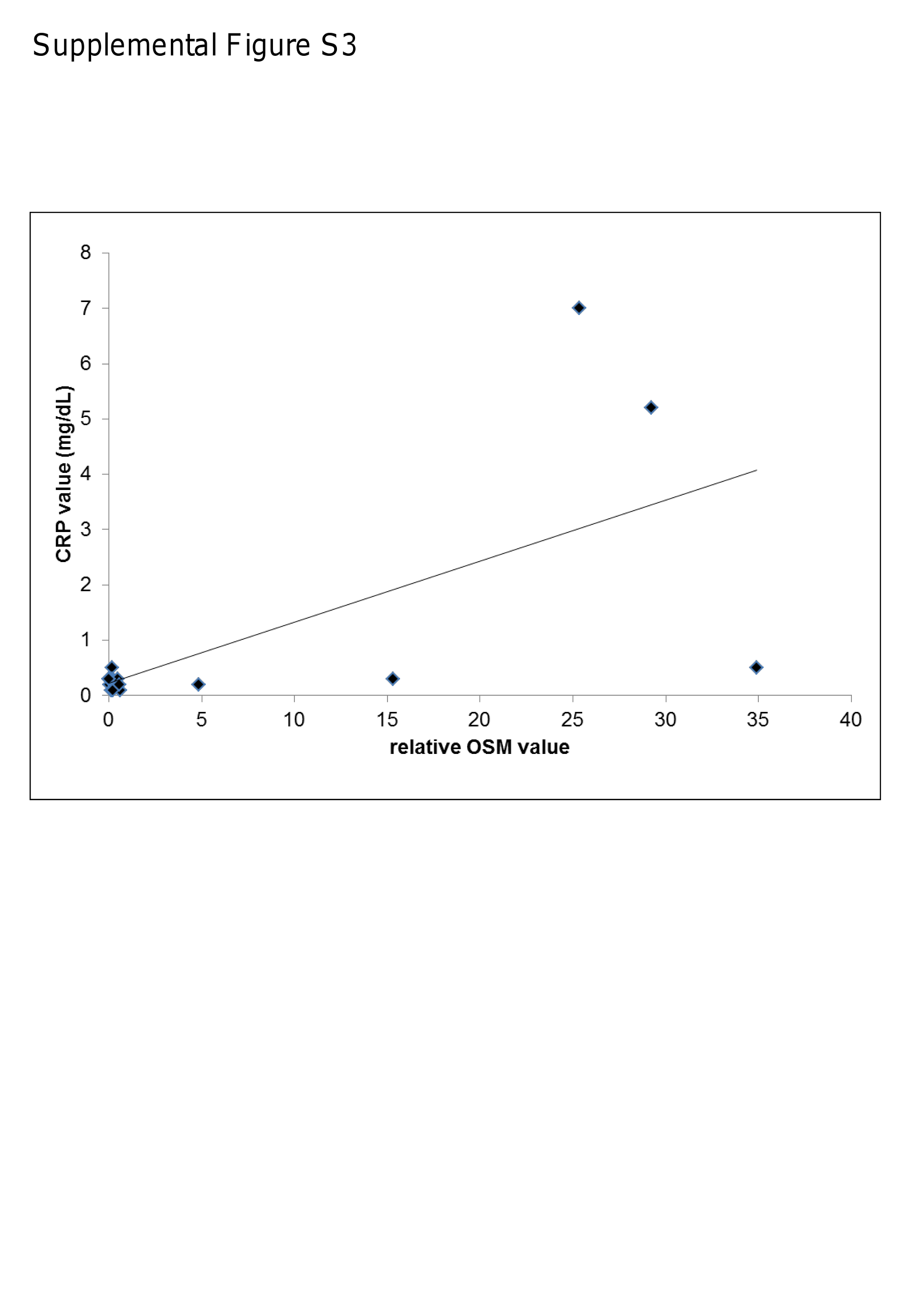

Supplement: Figure S3 — Correlation between CRP values (mg/dL) and arbitrary OSM values in inflamed lesions of patients with Crohn's disease (r = 0.66). (TIF) [file pone.0093498.s003.tif]
