# Supplementary material for: Oncostatin M Mediates STAT3-Dependent Intestinal Epithelial Restitution via Increased Cell Proliferation, Decreased Apoptosis and Upregulation of SERPIN Family Members
Source: PLoS One. 2014 Apr 7;9(4):e93498. doi: 10.1371/journal.pone.0093498 (PMC3977870; doi:10.1371/journal.pone.0093498)
Supplement: File S1 — includes the following: Table S1. Demographics of IBD biopsy patients (IHC). Table S2. Demographics of Crohn's patients (qPCR). Table S3. Demographics of ulcerative colitis patients (qPCR). (DOCX) [file pone.0093498.s004.docx]

**Supplemental Table S1. Demographics of IBD biopsy patients (IHC)**

| **Pat** | **Age** | **Sex** | **Type** | **Medication at biopsy** | **Mucosal healing** | **CDAI/CAI at biopsy** | **CRP (mg/dL)at biopsy** |
| --- | --- | --- | --- | --- | --- | --- | --- |
| 1 | 27 | m | active CD | none | no | CDAI 160 | 5.2 |
| 2 | 54 | f | active CD | ustekinumab | no | CDAI 159 | 4.7 |
| 3 | 53 | m | remission CD | steroids | yes | CDAI 0 | 0.2 |
| 4 | 36 | m | remission CD | anti-TNF | no | CDAI 72 | 2.8 |
| 5 | 44 | f | active UC | mesalazine, steroid enema | no | CAI 6 | 4.4 |
| 6 | 34 | m | active UC | mesalazine, steroid enema | no | CAI 5 | 1.2 |
| 7 | 61 | m | remission UC | mesalazine | no | CAI 0 | 0.3 |
| 8 | 39 | f | remission UC | no | yes | CAI 1 | 0.6 |

**Supplemental Table S2. Demographics of Crohn’s patients (qPCR)**

| **Pat** | **Age** | **Sex** | **Mean arbitrary OSM value inflamed lesion(s)** | **Mean arbitrary OSM value not inflamed lesion(s)** | **Medication at biopsy** | **CDAI at biopsy** | **CRP(mg/dL) at biopsy** |
| --- | --- | --- | --- | --- | --- | --- | --- |
| 1 | 38 | m | n/a | 0.03 | anti-TNF | 5 | 1.2 |
| 2 | 31 | f | 0.19 | 0.05 | anti-TNF | 3 | <0.1 |
| 3 | 21 | m | 4.86 | 0.02 | anti-TNF, steroids | 0 | 0.2 |
| 4 | 25 | m | 0.05 | 0.00 | anti-TNF | 37 | 0.1 |
| 5 | 36 | f | 0.58 | 0.12 | anti-TNF | 14 | 0.2 |
| 6 | 36 | f | 0.22 | 0.17 | anti-TNF | n/a | 0.3 |
| 7 | 43 | m | n/a | 0.14 | thiopurine | 0 | 0.1 |
| 8 | 30 | f | 0.00 | 0.09 | anti-TNF | 46 | 0.3 |
| 9 | 29 | m | n/a | 0.08 | anti-TNF | 7 | 1.0 |
| 10 | 26 | f | n/a | 0.17 | anti-TNF | 12 | <0.1 |
| 11 | 31 | m | n/a | 0.07 | anti-TNF | 94 | 0.1 |
| 12 | 21 | m | n/a | 0.10 | anti-TNF | 12 | <0.1 |
| 13 | 20 | m | n/a | 0.06 | anti-TNF | 4 | 0.2 |
| 14 | 26 | m | n/a | 0.00 | anti-TNF | 60 | 1.3 |
| 15 | 59 | m | n/a | 0.00 | anti-TNF | 32 | 0.6 |
| 16 | 42 | f | 0.00 | 0.00 | anti-TNF | 74 | 0.3 |
| 17 | 22 | m | n/a | 0.47 | anti-TNF | 0 | 0.1 |
| 18 | 22 | m | 29.22 | n/a | steroids | 154 | 5.2 |
| 19 | 37 | f | 34.93 | 0.07 | anti-TNF | 44 | 0.5 |
| 20 | 24 | m | 0.23 | n/a | anti-TNF, steroids | 250 | 0.3 |
| 21 | 44 | f | 0.31 | 0.02 | anti-TNF | 84 | <0.1 |
| 22 | 40 | m | n/a | 0.00 | anti-TNF | 0 | 2 |
| 23 | 30 | m | 20.34 | 0.17 | anti-TNF | 167 | 7.0 |

**Supplemental Table S3. Demographics of ulcerative colitis patients (qPCR)**

| **Pat** | **Age** | **Sex** | **Mean arbitrary OSM value inflamed lesion(s)** | **Mean arbitrary OSM value not inflamed lesion(s)** | **Medication at biopsy** | **CAI at biopsy** | **CRP (mg/dL)at biopsy** |
| --- | --- | --- | --- | --- | --- | --- | --- |
| 1 | 40 | f | n/a | 0.10 | thiopurine | 7 | 0.3 |
| 2 | 42 | m | n/a | 0.06 | anti-TNF | n/a | 0.1 |
| 3 | 25 | f | n/a | 0.00 | anti-TNF | n/a | <0.1 |
| 4 | 35 | f | 0.30 | 0.04 | n/a | 2 | 0.5 |
| 5 | 61 | m | 0.00 | 0.08 | anti-TNF | 0 | 0.3 |
| 6 | 31 | m | 0.02 | 0.06 | anti-TNF | 0 | 0.3 |
| 7 | 52 | m | 3.34 | 0.03 | thiopurine | 6 | 0.1 |
| 8 | 24 | m | 15.33 | 3.32 | anti-TNF | 7 | 0.2 |
| 9 | 25 | m | n/a | 0.00 | anti-TNF | 1 | 0.1 |
| 10 | 61 | m | 0.02 | 0.14 | thiopurine | 3 | 0.1 |
